# Red Tide Detection Method Based on Improved U-Net Model-Taking GOCI Data in East China Sea as an Example

**DOI:** 10.3390/s23229195

**Published:** 2023-11-15

**Authors:** Yanling Han, Tianhong Ding, Pengxia Cui, Xiaotong Wang, Bowen Zheng, Xiaojing Shen, Zhenling Ma, Yun Zhang, Haiyan Pan, Shuhu Yang

**Affiliations:** Key Laboratory of Fisheries Information, Shanghai Marine Intelligent Information and Navigation Remote Sensing Engineering Technology Research Center, Ministry of Agriculture, College of Information, Shanghai Ocean University, Shanghai 201306, China; ylhan@shou.edu.cn (Y.H.); 17335885009@163.com (T.D.); 15021865276@163.com (P.C.); wxt_7182@163.com (X.W.); zbw17377349137@163.com (B.Z.); zlma@shou.edu.cn (Z.M.); y-zhang@shou.edu.cn (Y.Z.); hy-pan@shou.edu.cn (H.P.); shyang@shou.edu.cn (S.Y.)

**Keywords:** red tide, GOCI, complex boundary, ECA, ASPC, U-Net

## Abstract

In the coastal areas of China, the eutrophication of seawater leads to the continuous occurrence of red tide, which has caused great damage to Marine fisheries and aquatic resources. Therefore, the detection and prediction of red tide have important research significance. The rapid development of optical remote sensing technology and deep-learning technology provides technical means for realizing large-scale and high-precision red tide detection. However, the difficulty of the accurate detection of red tide edges with complex boundaries limits the further improvement of red tide detection accuracy. In view of the above problems, this paper takes GOCI data in the East China Sea as an example and proposes an improved U-Net red tide detection method. In the improved U-Net method, NDVI was introduced to enhance the characteristic information of the red tide to improve the separability between the red tide and seawater. At the same time, the ECA channel attention mechanism was introduced to give different weights according to the influence of different bands on red tide detection, and the spectral characteristics of different channels were fully mined to further extract red tide characteristics. A shallow feature extraction module based on Atrous Spatial Pyramid Convolution (ASPC) was designed to improve the U-Net model. The red tide feature information in a multi-scale context was fused under multiple sampling rates to enhance the model’s ability to extract features at different scales. The problem of limited accuracy improvement in red tide edge detection with complex boundaries is solved via the fusion of deep and shallow features and multi-scale spatial features. Compared with other methods, the method proposed in this paper achieves better results and can detect red tide edges with complex boundaries, and the accuracy, precision, recall, and F1-score are 95.90%, 97.15%, 91.53%, and 0.94, respectively. In addition, the red tide detection experiments in other regions with relatively concentrated distribution also prove that the method has good applicability.

## 1. Introduction

Red tide refers to an ecological anomaly in which some microalgae, protozoa, or bacteria in the ocean proliferate or accumulate to a certain level under certain environmental conditions, causing water discoloration or harming other organisms in the ocean [1]. With the change in the coastal marine environment in China, the frequency of red tides is increasing, the area and economic loss caused by red tides are also increasing, and the negative impacts on the marine ecosystem, the coastal aquaculture industry, and tourism industry are increasing, and the toxins produced by red tide also pose a threat to human health [2]. In recent years, the red tide disaster has attracted more and more attention, and red tide detection has important research significance.

At present, there are three traditional methods for detecting red tide: “visual method”, “instrument method”, and “remote sensing method”. The first two methods require a significant amount of manpower and resources for red tide detection, and the harsh outdoor environment increases the difficulty of detection. Satellite remote sensing technology can effectively extract information from different dimensions, such as biological and environmental conditions at the time of red tide occurrence. It can better understand the characteristics of red tide and estimate the occurrence range, distribution area, and general development trend of red tide to provide an important means for the rapid synchronization, large-scale spatial, and high-frequency continuous monitoring of red tide. The remote sensing method for detecting red tide is mainly divided into three categories. The first category is to distinguish red tides by chlorophyll content and other parameters [3,4]. In 2018, Jiang Dejuan et al. used chlorophyll concentration and other parameters to visually identify red tides in the Bohai Sea [5]. The second category is to detect red tides through the obvious spectral differences in water bodies during the occurrence of red tides. During the occurrence of red tide, the sudden proliferation or aggregation of protozoa or bacteria leads to changes in the color and spectral characteristics of water bodies, especially in the green band (550 nm), with significant fluctuations. Red tide is detected using these spectral changes [6,7]. In 2017, Jiang Binbin et al. detected red tides using the normalized water emissivity (550 nm) obtained as the characteristic band [8]. The third type is to detect red tide using the fluorescence of algae, such as some algae exhibit fluorescence in the red light band. In 2018, Zhang Feng et al. extracted the red tide index using the fluorescence line height of different water bodies [9]. In 2021, Wang Siyuan et al. conducted red tide detection by selecting band information of 460, 530, 650, and 750 nm. However, the above methods usually need to determine the threshold value in advance to effectively diagnose red tide, and the threshold value is determined based on expert experience and prior knowledge, etc. Threshold-based red tide detection is inaccurate and difficult to apply globally due to regional dependence and sensitivity to environmental factors. Therefore, a threshold-free method is needed.

Deep learning has been widely used in remote sensing applications such as image classification, object recognition, and image fusion. Due to its powerful big data mining and feature extraction capabilities, deep learning has also been applied to red tide detection and achieved good results [10,11]. In 2018, Hu et al. proposed a CNN network composed of 8 fully connected layers, and the results proved that the accuracy of red tide detection was higher than that of traditional machine learning methods [12]. In 2019, Kim et al. used GOCI data and the U-Net network to study red tide detection in waters around the Korean Peninsula. U-Net was widely used in pixel classification or target segmentation and achieved good results in red tide detection [13]. In 2020, Lee et al. developed a novel red tide detection scheme for the southern coast of the Korean Peninsula using multi-layer feedforward neural networks based on deep learning and high spatio-temporal resolution satellites [14]. In 2022, Zhao et al. proposed the RDU-Net network based on the HY-1D coastal imager, which improved the detection accuracy of deep learning at the edge of red tides and scattered areas [15]. The above research verifies the feasibility of using deep learning for remote sensing red tide detection. However, the detection methods of red tide based on deep-learning models, such as the CNN model and other red tide detection methods show high detection accuracy in the concentrated areas of red tide, but the detection accuracy is not ideal in the scattered red tide, while the detection method based on U-Net model has a good performance in the detection of large-scale concentrated red tide and banded red tide. However, the detection accuracy of red tide edge for complex boundaries still needs to be improved. Based on the above research, this paper proposes a red tide detection method based on an improved U-Net model.

## 2. Data and Methodology

### 2.1. Satellite Data

**Figure 1:**
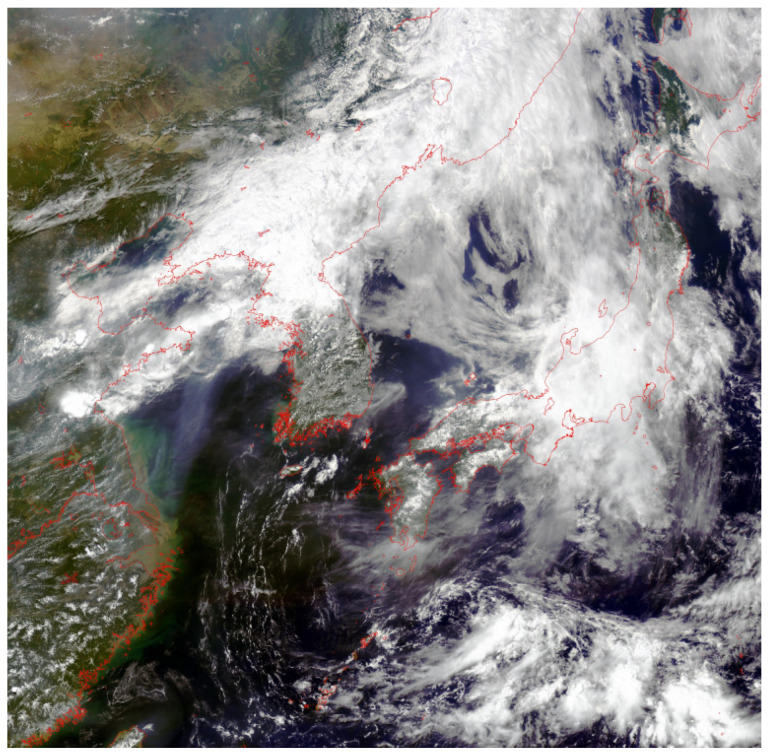
True color map of GOCI satellite coverage range.

At present, the main satellite sensors currently used include MODIS, GOCI, etc., among which the GOCI sensor can acquire 8 scene images with a time interval of 1 h every day, which is different from the polar orbiting satellite that can only pass the scene once every day, making it possible to timely monitor the marine environment and marine disaster by remote sensing. GOCI (Geostationary Ocean Color Imager) is a new-generation South Korean ocean color imager. It is carried on the first geostationary Meteorological Satellite COMS (Communication, Ocean, Meteorological Satellite) launched by South Korea in 2010; the coverage range is 2500 km × 2500 km, and its spatial resolution is 500 m. It has a very high time resolution, with eight hours of observation data every day from 8:30 to 15:30 local time. The time interval is 1 h, which is shown in Figure 1 as the true color map of the coverage area of the GOCI satellite. The spectral resolution is 402–885 nm and contains 8 band information, as shown in Table 1. GOCI data can be classified into L1B data, L2 data, and L3 data.

### 2.2. Data Set Construction

The data of this experiment are based on the red tide disaster information published in China Ocean Bulletin. From 24 July to 27 July 2016, the single largest area of red tide process caused by the red mesonuclium occurred in the sea area southeast of Zhujiajian, Zhoushan. We chose the L1B level data from 26 July during the outbreak of red tide in the East China Sea area, where there is less cloud cover. For the GOCI reflectance values, the Geographic Lat/Lon projection was used to generate the required Geographic Lookup Table (GLT) file from the longitude and latitude information of GOCI, and GLT geometric correction was performed using ENVI software (ENVI 5.6). Atmospheric correction is performed using the GOCI data atmospheric correction method built into SeaDas, officially published by NASA OBPG. Seadas was used to carry out the atmospheric correction [16].

**Figure 2:**
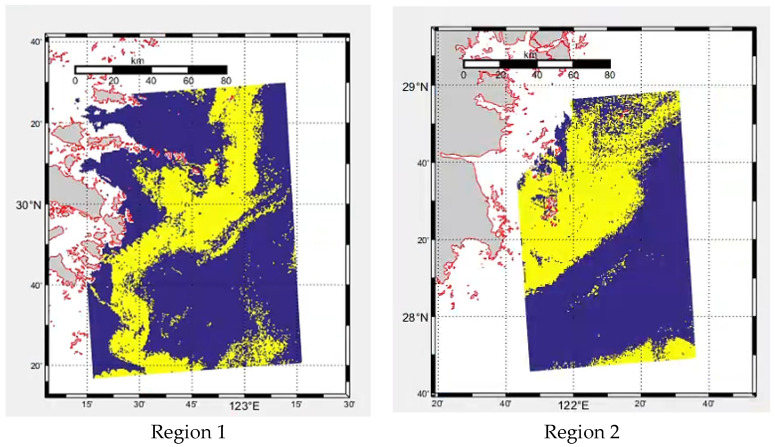
Visual results of chlorophyll concentration products from GOCI data. 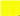 Red tide; 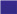 Seawater; 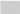 Land; 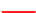 Coastline; 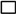 Unstudied region.

For the atmospheric corrected data, the range of red tide occurrence was first determined according to chlorophyll products. After determining the occurrence range of red tide, Regions 1 and 2 with complete data were selected to make samples. Region 1 is mainly used for the design and verification of the model. Due to the problem of large-area cloud cover in some time periods and considering sample balance, remote sensing data at 9:30, 10:30, 11:30, and 12:30 in Region 1 are finally selected as training samples, and remote sensing data at 13:30 are selected as test samples. Region 2 is mainly used to explore the applicability of the model. Remote sensing data at 8:30, 9:30, 10:30, and 11:30 in Region 2 are selected as training samples, and remote sensing data at 14:30 are selected as test samples. Figure 2 shows the actual occurrence range of red tides in Regions 1 and 2. In addition, taking into account computer memory and speed, the input sample size of the model is set to 32 × 32. Therefore, the images and corresponding labels are randomly divided into 32 × 32-pixel samples.

### 2.3. Relevant Methods

#### 2.3.1. U-Net Model

U-Net network is a kind of convolutional neural network widely used in image segmentation tasks, proposed by Ronneberger et al. in 2015 [17]. As a pixel-level prediction model that can be trained end-to-end, the structure of the network has the advantage of supporting a small amount of data to train the model and can better handle the small sample image segmentation task. In remote sensing data processing, spatial resolution is usually represented by pixel size. In this experiment, pixel point refers to the ground space range of 500 × 500 m. The encoding and decoding structure of U-Net’s jump connection can integrate features of different levels and obtain higher segmentation accuracy via pixel-level classification. Therefore, the red tide detection research is carried out on the basis of U-Net model.

The overall network structure of U-Net can be divided into two parts, that is, the down-sampling network and the up-sampling network. The down-sampling network reduces the resolution and size of the image via continuous convolution and pooling layers, while the up-sampling network restores the low-resolution image to the original image size via deconvolution and skip connections. Based on the traditional coding-decoding structure, the idea of long connection is introduced, which makes the shallow local features reused in the deep layer of the network and greatly improves the accuracy of semantic segmentation. Down-sampling is a typical convolutional neural network structure, which adopts the structure of two convolutional layers and one maximum pooling layer repeatedly, and the dimension of the feature graph is doubled after each pooling operation. First, deconvolution operation is performed once in the upper sampling to halve the dimension of the feature map; then, the feature map obtained from the corresponding compression channel is spliced to form a feature map of twice the size; then, two convolutional layers are used for feature extraction, and this structure is repeated; in the final output layer, two convolution layers are used to map the 64-dimensional feature map to the 2-dimensional output map, and each pixel is assigned a category label whether it is a red tide or not. Among them, feature map is the feature expression extracted from the original input image in the process of deep learning. It is the intermediate result obtained via a series of convolution check input images. Features at all levels are obtained using the red tide detection model, and red tide detection is finally realized.

#### 2.3.2. Comparison Method

For comparison, this study selected a full convolutional neural network (FCN) and a machine learning method, support vector machine (SVM). The details of each algorithm are as follows:

Fully convolutional networks (FCNs) [18], a framework for image semantic segmentation proposed by Jonathan Long et al. in 2015, is the first work of deep learning in the field of semantic segmentation. FCN replaces the fully connected layer behind the traditional CNN with a convolutional layer. At the same time, in order to reduce the image size caused by convolution and pooling, the up-sampling method is used to restore the image size. The FCN network structure is mainly divided into two parts: the full convolution part and the deconvolution part. The full convolution part is used to extract features, and the deconvolution part is used to get the semantic segmentation image of full size by up-sampling. FCN consists of three networks: FCN–32s, FCN–16s, and FCN–8s. FCN–32s recovers the input size from the feature map with 32 times down-sampling, and FCN–16s and FCN–8s recover the input size from 16 times and 8 times down-sampling. The more FCN–8s integrate maximum pooling layer information features and use deconvolution layer for up-sampling, the finer the segmentation effect. Therefore, FCN–8s is superior to FCN–16s and FCN–32s in image segmentation, and FCN–8s is chosen for comparison experiments [19].

SVM is a kernel-based supervised classification algorithm proposed by Cortes and Vapnik [20]. SVM is mainly a method for the learning, classification, and prediction of small sample data and has good generalization ability. It has proven to be a powerful machine learning algorithm for pattern recognition and nonlinear regression, with great advantages in the field of classification. The basic idea of SVM is to find the optimal hyperplane that can correctly divide the training data set with maximum geometric spacing. One side of the hyperplane is red tide, and the other side is non-red tide [21].

## 3. Improved U-Net Red Tide Detection Model

### 3.1. Red Tide Detection Flow Chart Based on Improved U-Net Model

**Figure 3:**
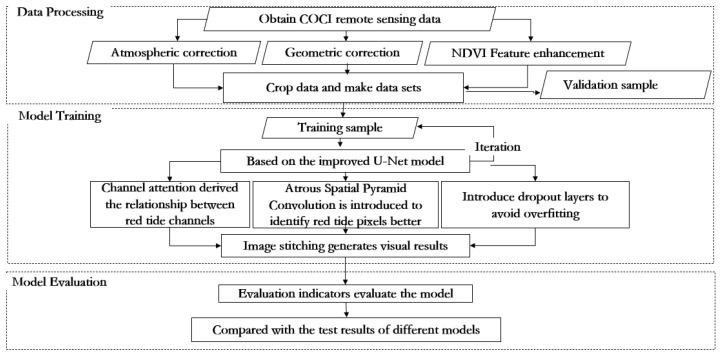
Overall flow diagram of the improved U-Net model.

The overall flow diagram of this paper is shown in Figure 3, which is mainly divided into three parts: data processing, model training, and model evaluation. Firstly, the L1B data of the GOCI satellite was preprocessed via geometric correction, atmospheric correction, and other data processing methods. The red tide feature was enhanced by integrating NDVI features, and then, the data was clipped to make a data set. Model training is to input the processed data set into the improved U-Net red tide detection model for training. Based on the traditional U-Net, the improved U-Net introduces the ECA channel attention mechanism module, Atrous Spatial Pyramid Convolution (ASPC), and dropout layer so that the model can effectively realize cross-channel interaction and assigns different weights according to the influence degree of different channels. The spectral features of different channels were fully mined to further extract the red tide features in depth, and the red tide feature information in a multi-scale context was aggregated under multiple sampling rates. Through the fusion of deep and shallow features and multi-scale spatial features, red tide at complex boundaries could be better detected. After the model was trained, relevant parameters were saved. Finally, accuracy and precision were used to evaluate the detection effect of the model and were compared with other red tide detection methods.

### 3.2. Feature Enhancement-NDVI

High-resolution remote sensing images contain more complex red tide characteristic information, but due to the limitation of spatial resolution and the small spectral difference between pixels at the red tide boundary, it is difficult to distinguish the difference between turbids and red tide boundaries, so the separability of spectral information among turbids, red tides, and seawater around the red tide boundary is weak. It can be seen from the characteristic information of the selected red tide samples that the red tide responds strongly in the red and near-infrared bands. Therefore, NDVI was introduced to enhance the characteristic information of red tide. In the GOCI band data, the red and near-infrared bands correspond to Band 5 and Band 8, respectively. The NDVI Formula (1) is as follows:

(1) $$\mathrm{NDVI}=\frac{R_{\mathrm{rs}}\left(865\right)-R_{\mathrm{rs}}\left(660\right)}{R_{\mathrm{rs}}\left(865\right)+R_{\mathrm{rs}}\left(660\right)}$$

### 3.3. Attention Mechanism of ECA Channel

Studies have shown that there must be red tides in the high chlorophyll sea area, and there may be red tides in the low chlorophyll sea area. When the chlorophyll content in the water increases, the red tide will also break out, and the blue-green band value in the remote sensing image will also have a relatively large change. Therefore, in red tide detection, different weights are given according to the different importance of different bands to red tide detection, which can effectively improve the classification accuracy of the detection model.

**Figure 4:**
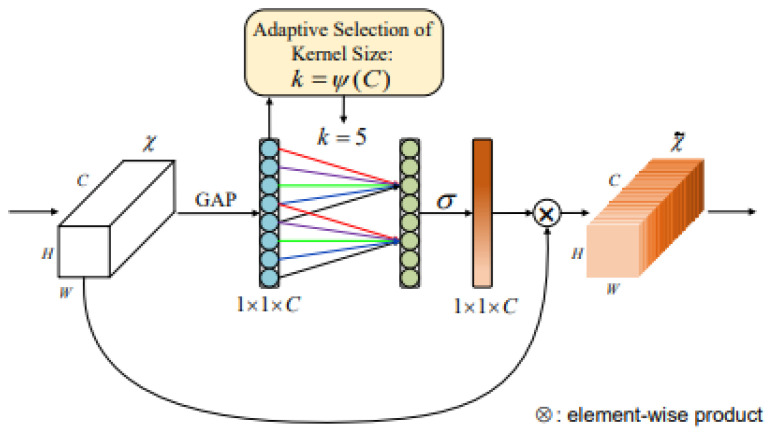
ECA module.

In the pooling convolution process of traditional network model training, it is usually the default that each channel of the feature graph has the same weight, but in the actual situation, different channels of the feature graph have different degrees of influence on feature extraction. If the weight of each channel is the same, it will result in a certain degree of information loss. ECA module can effectively realize cross-channel interaction, give different weights according to the influence degree of different channels, and fully excavate the spectral characteristics of different channels to further extract red tide characteristics to improve the classification accuracy of the detection model. Here, fully excavating means extracting the characteristic information of red tide by channel weighting based on the sensitivity of different channels to red tide detection. Suppose that a feature graph of H × W × C is input, a feature graph of 1 × 1 × C is obtained via global pooling (H × W is the pooling size), and only the channel dimension is retained. Then, a 1D convolution layer is connected so that the channel of each layer has information interaction with the channel of the adjacent layer. The weight is shared, and then, a Sigmod layer is connected to obtain the features of 1 × 1 × C; finally, the original H × W × C and 1 × 1 × C feature maps are fully multiplied to obtain the feature map after increasing the attention weight. With a full multiplication of the feature map, the attention weight can be increased, the correlation between different channels in the feature map can be adjusted, and the accuracy of the model can be improved [22]. The specific structure is shown in Figure 4:

### 3.4. Atrous Spatial Pyramid Pooling (ASPP)

**Figure 5:**
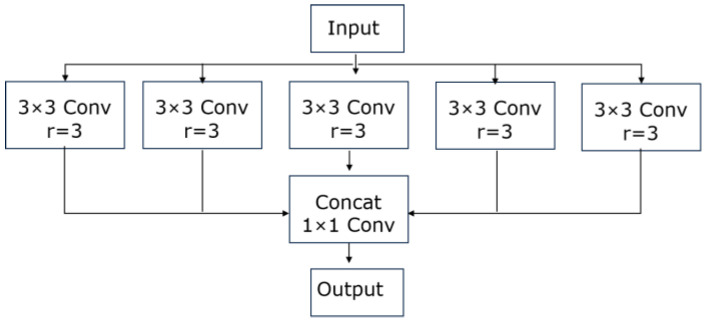
ASPP module.

**Figure 6:**
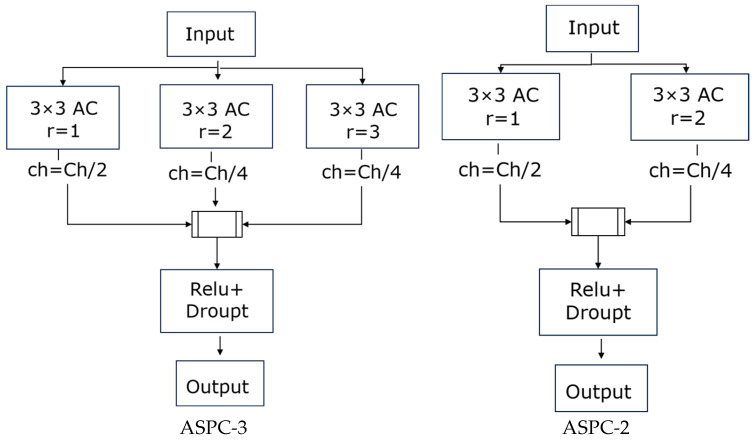
Improved ASPP module.

Atrous Spatial Pyramid Pooling (ASPP) samples the given input in parallel with cavity convolution at different sampling rates to realize pooling operation and then parallels it with global average pooling to form a new feature pyramid model to aggregate multi-scale context information, which combines atrous convolution to expand the sensitivity field of the convolution kernel without down-sampling [23]. Atrous convolution with expansion rates of 1, 3, and 5 and convolution kernel size of 3 × 3 is used, and depth-separable convolution is used to reduce the number of parameters. The concrete implementation of the ASPP module: the first branch is 1 × 1 standard convolution, and the purpose is to maintain the original receptive field; the second to fourth branches are depth-separable convolutions with different expansion rates, aiming at feature extraction to obtain different receptive fields; the fifth branch is to pool inputs globally to obtain global features; finally, the feature graphs of the five branches are stacked on the channel dimension, and the information of different scales is fused via 1 × 1 standard convolution. Figure 5 shows the structure:

In this paper, Atrous Spatial Pyramid Convolution (ASPC) is proposed based on the idea of ASPP. ASPC uses multiple convolutions with different expansion rates in parallel to form a new feature pyramid model to enlarge the receptive field of the convolution kernel, which aggregates multi-scale context information and enhances the ability of the model to extract features of different scales to better detect red tides at complex boundaries. In this paper, a three-way cavity convolution parallel structure is adopted, in which the expansion rate of ASPC-3 is 1, 2, and 3, respectively, and the number of channels is 50%, 25%, and 25%, respectively. The expansion rate of ASPC-2 was 1 and 2, and the proportion of channels was 50% and 50%, respectively. It can be seen from the calculation that according to the convolutional parallel structure with expansion rates of 1, 2, and 3, the length of the ASPC receptive field presents a linear increase trend with a slope of 3 as the number of convolutional layers increases. When the expansion rate is 2 and the size of the convolutional kernel is 3 × 3, the receptive field increases to 7 × 7. When the expansion rate is 3, the size of the convolution kernel is 3 × 3, and the receptive field is increased to 11 × 11, the red tide feature information in a multi-scale context can be aggregated. The specific structure is shown in Figure 6.

### 3.5. Improved U-Net Model Framework

**Figure 7:**
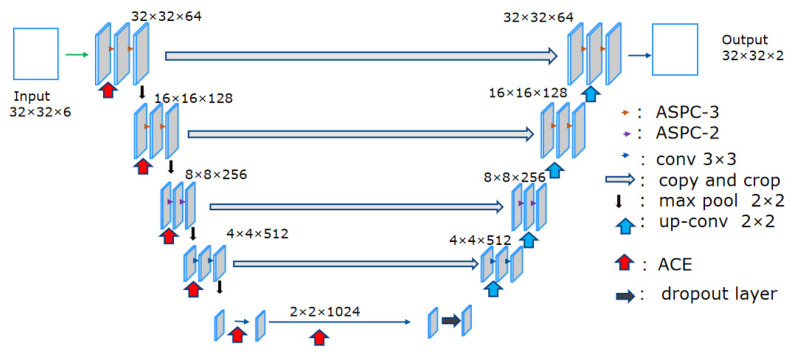
Improved U-Net model structure.

Due to the limitation of band and spectral resolution, the detection of red tide in medium and high-resolution satellite images requires strong feature information, especially for the small and dispersed banded red tide in the East China Sea. In addition, the traditional U-Net cannot effectively extract detailed information about red tide, and there is a larger error at the boundary of red tide compared with the concentrated area. Therefore, on the basis of the traditional U-Net, the ECA module is introduced to give different weights according to the influence degree of different bands, and the spectral characteristics of different channels are fully mined to further extract red tide characteristics, thus improving the classification accuracy of the detection model. At the same time, the common convolution blocks of the first two layers in the U-Net structure diagram are replaced with the ASPC-3 module, and the common convolution blocks of the third layer in the U-Net structure diagram are replaced with the ASPC-2 module. As shown in Figure 7, red tide feature information in a multi-scale context can be aggregated under multiple sampling rates to better detect red tide at complex boundaries, thus improving the classification accuracy of the detection model. In addition, since the model training in this study is based on a small training set, the information lost in down-sampling is reduced to some extent by adding a dropout layer before up-sampling to avoid over-fitting. The overall structure of the improved red tide detection model in this paper is shown in Figure 7.

## 4. Experimental Results and Analysis

### 4.1. Experimental Settings

The experiment in this paper was conducted on the experimental equipment configured with Inter core i7-11700, 2.50 GHz processor, and 16 G RAM1, and the overall experiment was implemented based on python and tensorflow frameworks. For the improved U-Net model, the input size is 32 × 32, the number of channels is 7, and the output is a classified image of the same size. In this experiment, the Adam algorithm is used to optimize the model [24]. The batch size, dropout rate, and training times are 50, 0.3, and 120, respectively.

### 4.2. Model Evaluation Index

In order to evaluate the classification accuracy of the model more comprehensively, this paper introduces accuracy, precision, recall, and F1-score as evaluating indexes. The accuracy represents the proportion of the number of samples in which positive samples are classified as positive and negative samples are classified as negative in all samples. Precision is the ratio of accurately detected red tide pixels to all identified red tide pixels. The recall represents the percentage of pixels classified as red tides that are correctly classified. F1-score is an indicator of the accuracy of the red tide detection method, which combines precision and recall. The calculation methods are given below:

(2) $$\mathrm{Accuracy}=\frac{\mathrm{TP}+\mathrm{TN}}{\mathrm{TP}+\mathrm{TN}+\mathrm{FP}+\mathrm{FN}}\times 100\%$$

(3) $$\mathrm{Precision}=\frac{\mathrm{TP}}{\mathrm{TP}+\mathrm{FP}}\times 100\%$$

(4) $$\mathrm{Recall}=\frac{\mathrm{TP}}{\mathrm{TP}+\mathrm{FN}}\times 100\%$$

(5) $$F1-\mathrm{score}=2\times \frac{\mathrm{Precision}\times \mathrm{Recall}}{\mathrm{Precision}+\mathrm{Recall}}$$

### 4.3. Experimental Results

Due to the small spectral difference between the pixels in the boundary part, the separability of red tide and seawater is weak, and it is difficult to accurately distinguish the complex boundary of red tide with small scale and dispersed distribution. However, red tides respond strongly in the red and near-infrared bands, and NDVI is introduced to enhance the characteristic information of red tides so that it is easier to distinguish the small-scale and dispersed banded boundary red tides. Red tide classification was compared between the original six-band GOCI data and the data with NDVI feature enhancement based on the original six-band data on the basis of the basic U-Net model. Use the training and test sets made in Section 2.2. Table 2 summarizes the detection accuracy of red tide with different characteristic data. As shown in Table 2, after the introduction of NDVI feature enhancement on the basis of the original six-band data, the detection accuracy of each test set has been improved, in which the accuracy, precision, recall, and F1-score have increased by 1.52%, 2.19%, 2.19%, and 0.03, respectively. The experimental results also verified the difference between red tide NDVI and seawater NDVI. It has a significant detection effect on small-scale and dispersed banded red tides.

When the red tide breaks out, the blue-green band data in the remote sensing image will also have a relatively large change. On the basis of the traditional U-Net, the introduction of the ECA channel attention mechanism can effectively realize cross-channel interaction and give different weights according to the influence degree of different bands so that the model can pay maximum attention to the channel, which it needs to pay attention to, and fully explore the spectral characteristics of different channels to further extract red tide characteristics. It has a good recognition effect on the detection of complex boundary red tides with small-scale and dispersed strips, which can improve the classification accuracy of the detection model. Table 3 shows the experimental results of feature enhancement with the introduction of NDVI on the basis of the original six-band features and summarizes the accuracy of red tide detection with the introduction of ECA channel attention mechanism and Atrous Spatial Pyramid Convolution (ASPC) on the basis of traditional U-Net. As shown in Table 3, after the introduction of ECA on the basis of traditional U-Net, the detection accuracy of each test set is improved, among which the accuracy, recall, and F1-score are increased by 5.08%, 17.32%, and 0.12, respectively. The experimental results show that the attention mechanism of the ECA channel can effectively improve the detection effect of small-scale and dispersed banded red tide.

Atrous Spatial Pyramid Convolution (ASPC) is a multi-channel convolution with different expansion rates to expand the sensitivity field of the convolution kernel, aggregate the red tide feature information in a multi-scale context under multiple sampling rates, enhance the ability of the model to extract features of different scales, and better detect small and dispersed strips of complex boundary red tides, thus improving the detection effect of the model. On the basis of introducing the ECA channel attention mechanism into traditional U-Net, Atrous Spatial Pyramid Convolution (ASPC) is added. The common convolution blocks of the first two layers in the U-Net structure diagram are replaced with the ASPC-3 module, and the common convolution blocks of the third layer are replaced with the ASPC-2 module. Red tide pixels can be identified better via the effective fusion of spatial features of different scales, and higher detection accuracy is obtained. As shown in Table 3, the accuracy of each test set has been improved, in which the accuracy, precision, recall, and F1-score have increased by 4.54%, 1.46%, 16.92%, and 0.10, respectively. The experimental results show that cavity convolution can significantly improve the detection efficiency of small-scale and dispersed banded red tide.

The improved U-Net method is compared with the basic U-Net method, full convolutional neural network (FCN), and support vector machine (SVM) by introducing NDVI feature-enhanced data from the original six-band data. The parameters of the full convolutional neural network used in this experiment are the same as those of the improved U-Net. Table 4 summarizes the accuracy of these methods for red tide detection. Compared with the shallow feature extraction of SVM and FCN–8s, the encoding and decoding structure of U-Net’s jump connection can integrate features of different levels and obtain higher segmentation accuracy by classifying each pixel point. As shown in Table 4, with the same data set and parameters, U-Net outperformed SVM and FCN–8s with an accuracy of 86.28%. The improved U-Net method introduces the ECA module and gives different weights according to the influence degree of different bands to distinguish and utilize the characteristic information of different bands conducive to red tide detection to the greatest extent. The common convolution blocks of the first three layers in the U-Net structure diagram are replaced with Atrous Spatial Pyramid Convolution (ASPC). The characteristic information of red tide in a multi-scale context is effectively fused under the condition of multiple sampling rates, and the detection accuracy of red tide is higher. As shown in Table 4, the performance of the improved U-Net model is better than that of the basic U-Net model. The accuracy, precision, recall, and F1-score of the test set are increased by 9.62%, 1.48%, 34.24%, and 0.22, respectively. The experimental results show that the improved U-Net method is more suitable for red tide detection.

**Figure 8:**
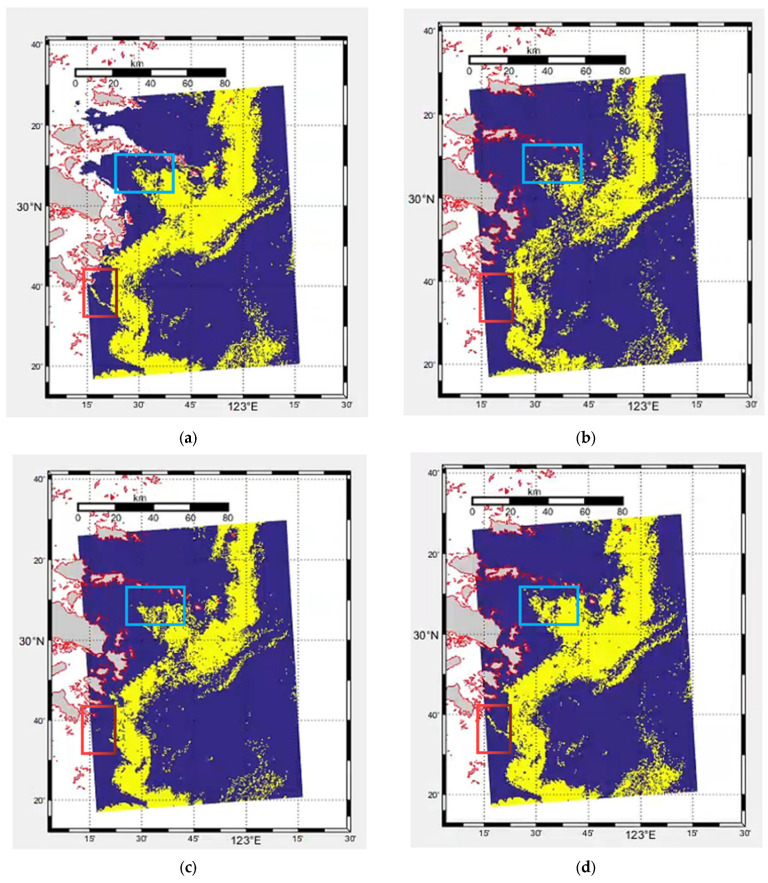
Region 1 red tide detection results based on different improved methods. (**a**) Actual red tide occurrence area, (**b**) Detection result with original six-band data, (**c**) Detection result with NDVI feature enhancement, (**d**) Detection result with the basic U-Net + ECA + ASPC. 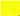 Red tide; 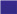 Seawater; 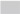 Land; 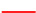 Coastline; 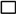 Unstudied region.

Figure 8 shows the visualization of L2A chlorophyll product data in GOCI data, the result of the original six band data based on the basic U-Net model, the result of NDVI feature enhancement based on the basic U-Net model, the result of NDVI feature enhancement and the result of the improved U-Net model (U-Net + ECA + ASPC). The visual detection results shown in the figure further validate that the improved U-Net model can effectively improve the detection effect of red tide, especially for the red tide boundary regions that are difficult to accurately detect (for example, the blue and red marked areas in the figure).

### 4.4. Method Applicability Analysis

In order to explore the applicability of the enhanced NDVI data and the improved U-Net method for red tide detection in other regions, the method was applied to Region 2. The experimental environment and parameter settings in Region 2 are the same as those in Region 1.

Table 5 shows the red tide detection accuracy of the basic U-Net model based on the original six-band features of GOCI and the introduction of NDVI feature enhancement based on the original six-band features. As shown in 4–4, on the basis of the original six-band data, the separation of spectral information of red tide and seawater in turbidity water is improved after the introduction of NDVI feature enhancement, and high detection accuracy is achieved, in which accuracy, precision, recall, and F1-score are increased by 1.45%, 18.54%, 4.90%, and 0.11, respectively. The experimental results also verified the difference between red tide NDVI and seawater NDVI and also had a significant effect on the detection of red tide in a large range of concentrated patches, which proved that NDVI features had strong applicability to the detection of different types of red tide.

Table 6 shows the comparative analysis results of the improved U-Net method with the basic U-Net method, FCN, and SVM after feature enhancement by NDVI in Region 2. As shown in Table 6, with the same data set and parameters, U-Net outperformed SVM and FCN–8s with an accuracy of 87.56%. The performance of the improved U-Net model is better than that of the basic U-NET model, and the accuracy, precision, recall, and F1-score of the test set are increased by 4.49%, 2.07%, 8.34%, and 0.06, respectively. The experimental results show that compared with other methods, the improved U-Net method shows the best red tide detection effect, and the method is also suitable for the detection of large-scale block-distributed red tides in other areas.

**Figure 9:**
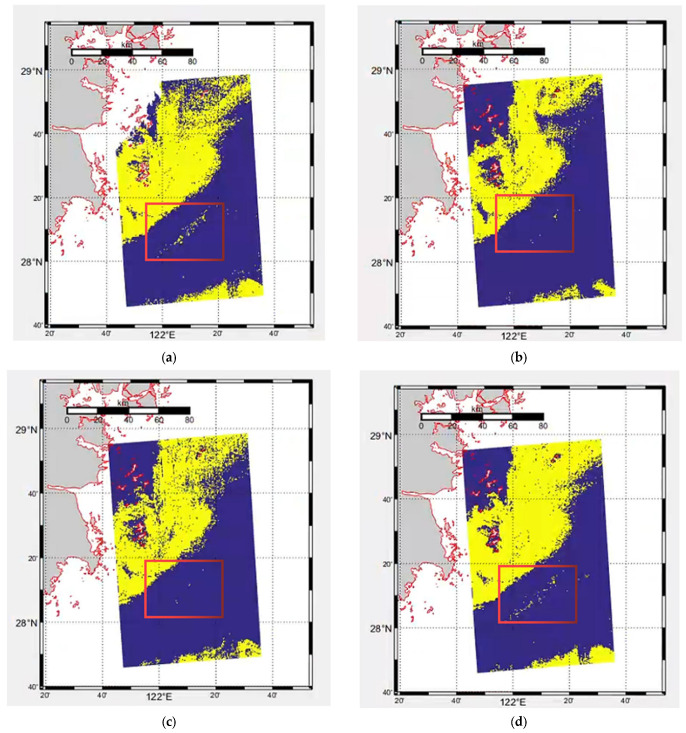
Region 2 red tide detection results based on different methods. (**a**) Actual red tide occurrence area, (**b**) Detection result with original six-band data, (**c**) Detection result with enhanced features, (**d**) Detection result with the improved U-Net. 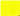 Red tide; 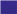 Seawater; 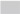 Land; 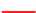 Coastline; 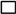 Unstudied region.

Based on the traditional U-Net, the improved U-Net method introduces an ECA module to maximize the network’s attention to the channels to which it needs to pay attention, fully mining the spectral characteristics of different channels to further extract red tide characteristics. At the same time, the common convolution blocks of the first three layers in the U-Net structure diagram are replaced with Atrous Spatial Pyramid Convolution (ASPC). Under the condition of multiple sampling rates, the contextual red tide feature information is generated, and the red tide with complex boundaries can be better detected through the fusion of deep and shallow features and multi-scale spatial features. Figure 9 shows the visualization of the actual red tide occurrence area, the predicted result with original six-band data, the predicted result with enhanced features, and that with the improved U-Net, respectively. This is further validated by the visual detection results shown, the improved U-Net can effectively detect the red tide, regardless of the concentrated distribution of red tide areas or the scattered distribution of red tide areas (such as the red marked area in region 2).

## 5. Conclusions

In order to further improve the accuracy of remote sensing red tide detection, this paper takes the red tide detection of GOCI remote sensing data in the East China Sea as an example. Based on the six-band data of GOCI, NDVI is introduced to enhance the characteristic information of red tide to improve the separability of red tide and seawater. An improved U-Net red tide detection method is proposed. Based on the attention mechanism of ECA channels, the spectral features of different channels were fully mined to further extract red tide characteristics. At the same time, a shallow feature extraction module based on Atrous Spatial Pyramid Convolution (ASPC) was designed, and multi-scale red tide feature fusion was used to enhance the feature extraction capability of the model to better identify red tide pixels with complex boundaries and improve the classification accuracy of the detection model. This study discusses the application of GOCI remote sensing data in red tide detection. The experimental results show that the proposed method has a good detection effect and provides a new reference for red tide detection methods.

## Figures and Tables

**Table 1:** Average solar irradiance outside the atmosphere at each band of GOCI.

Band Center (nm)	Solar Irradiance $\left(W\cdot m^{-2}\cdot \mu m^{-1}\cdot sr^{-1}\right)$
412	1738.8
443	1922.1
490	1988.4
555	1869.9
660	1535.3
680	1508.3
745	1295.9
865	967.6

**Table 2:** Red tide detection accuracy of different characteristic data.

Data	Accuracy/(%)	Precision/(%)	Recall/(%)	F1-Score
Original six-band features	84.76	93.48	55.10	0.69
Features enhanced with NDVI	86.28	95.67	57.29	0.72

**Table 3:** U-Net introduces different methods of red tide detection accuracy.

Method	Accuracy/(%)	Precision/(%)	Recall/(%)	F1-Score
Basic U-Net	86.28	95.67	57.29	0.72
U-Net + ECA	91.36	95.69	74.61	0.84
U-Net + ECA + ASPC	95.90	97.15	91.53	0.94

**Table 4:** Red tide detection accuracy of different methods.

Method	Accuracy/(%)	Precision/(%)	Recall/(%)	F1-Score
SVM	78.56	92.21	43.64	0.59
FCN-8s	82.27	93.09	44.25	0.60
U-Net	86.28	95.67	57.29	0.72
Improved U-Net	95.90	97.15	91.53	0.94

**Table 5:** Red tide detection accuracy of different characteristic data.

Data	Accuracy/(%)	Precision/(%)	Recall/(%)	F1-Score
Original six-band features	86.11	71.40	73.37	0.72
Features enhanced with NDVI	87.56	89.94	78.27	0.83

**Table 6:** Red tide detection accuracy of different methods.

Method	Accuracy/(%)	Precision/(%)	Recall/(%)	F1-Score
SVM	83.34	91.73	55.67	0.69
FCN-8s	85.64	88.14	70.77	0.78
U-Net	87.56	89.94	78.27	0.83
Improved U-Net	92.05	92.01	86.61	0.89

## Data Availability

Enquiries regarding the experimental data should be made by contacting the first author.

## References

[1] Hu C., Feng L. (2017). Modified MODIS fluorescence line height data product to improve image interpretation for red tide monitoring in the eastern Gulf of Mexico. J. Appl. Remote Sens..

[2] Zuo S., Li B. (2008). Marine Disasters Characteristics and Its Prevention Measures in China over the Past 20 Years. Meteorol. Disaster Reduct. Res..

[3] Noh J.H., Kim W., Son S.H., Ahn J., Park Y. (2018). Remote quantification of *Cochlodinium polykrikoides* blooms occurring in the East Sea using geostationary ocean color imager (GOCI). Harmful Algae.

[4] Ma L., Liu Y., Zhang B., Lu L., Sun G., Wang D., Liu Z., Li B., Wang Y., Zhang Y. (2021). Remotely sensed short-term changes in noctilucent algae blooms in the Bohai Sea. Int. J. Remote Sens..

[5] Jiang D., Zhang H. (2018). Analysis of spatial and temporal characteristics of chlorophyll-a concentration and red tide monitoring in Boha. Mar. Sci..

[6] Lee M.-S., Park K.-A., Micheli F. (2021). Derivation of Red Tide Index and Density Using Geostationary Ocean Color Imager (GOCI) Data. Remote Sens..

[7] Liu R.-J., Zhang J., Cui B.-G., Ma Y., Song P.-J., An J.-B. (2019). Red Tide Detection Based on High Spatial Resolution Broad Band Satellite Data: A Case Study of GF-1. J. Coast. Res..

[8] Jiang B., Li H., Teng G., Yan J., Zhang B. (2017). Using GOCI extracting information of red tide for time-series analysing in East China Sea. J. Zhejiang Univ..

[9] Feng Z., Xuying Y., Xiaoxiao S., Du Zhenhong Renyi L. Developing Process Detection of Red Tide Based on Multi-Temporal GOCI Images. Proceedings of the 2018 10th IAPR Workshop on Pattern Recognition in Remote Sensing (PRRS).

[10] Hill P.R., Kumar A., Temimi M., Bull D.R. (2020). HABNet: Machine Learning, Remote Sensing-Based Detection of Harmful Algal Blooms. IEEE J. Sel. Top. Appl. Earth Obs. Remote Sens..

[11] Zhu X.X., Tuia D., Mou L., Xia G., Zhang L., Xu F., Fraundorfer F. (2017). Deep Learning in Remote Sensing: A Comprehensive Review and List of Resources. IEEE Geosci. Remote Sens. Mag..

[12] Hu Y., Yi M., An J. (2018). Research on high accuracy detection of red tide hyperspecrral based on deep learning cnn. Int. Arch. Photogramm. Remote Sens. Spat. Inf. Sci..

[13] Kim S.M., Shin J., Baek S., Ryu J. (2019). U-Net Convolutional Neural Network Model for Deep Red Tide Learning Using GOCI. J. Coast. Res..

[14] Lee M.-S., Park K.-A., Chae J., Park J.-E., Lee J.-S., Lee J.-H. (2020). Red tide detection using deep learning and high-spatial resolution optical satellite imagery. Int. J. Remote Sens..

[15] Zhao X., Liu R., Ma Y., Xiao Y., Ding J., Liu J., Wang Q. (2022). Red Tide Detection Method for HY–1D Coastal Zone Imager Based on U–Net Convolutional Neural Network. Remote Sens..

[16] Shi F., Liu M., Zhou W. (2006). Study on radiation atmospheric correction method for second class water body using Sea WiFS data. J. Inn. Mong. Norm. Univ..

[17] Saood A., Hatem I. (2021). COVID-19 lung CT image segmentation using deep learning methods: U-Net versus SegNet. BMC Med. Imaging.

[18] Lu H., Liu Q., Liu X., Zhang Y. (2021). A Survey of Semantic Construction and Application of Satellite Remote Sensing Images and Data. J. Organ. End User Comput..

[19] Badrinarayanan V., Kendall A., Cipolla R. (2017). Segnet: A deep convolutional encoder-decoder architecture for image segmentation. IEEE Trans. Pattern Anal. Mach. Intell..

[20] Vapnik N.V. (1999). An overview of statistical learning theory. IEEE Trans. Neural Netw..

[21] Cho H.G.K.J. (2015). Prediction of effluent concentration in a wastewater treatment plant using machine learning models. J. Environ. Sci..

[22] Wang Q., Wu B., Zhu P., Li P., Zuo W., Hu Q. ECA-Net: Efficient Channel Attention for Deep Convolutional Neural Networks. Proceedings of the 2020 IEEE/CVF Conference on Computer Vision and Pattern Recognition (CVPR).

[23] He K., Zhang X., Ren S., Sun J. (2015). Spatial pyramid pooling in deep convolutional networks for visual recognition. IEEE Transactions on Pattern Analysis & Machine Intelligence.

[24] Kingma D., Ba J. (2014). Adam: A method for stochastic optimization. arXiv.

